# Preclinical Toxicological Assessment of A Novel siRNA, SLN360, Targeting Elevated Lipoprotein (a) in Cardiovascular Disease

**DOI:** 10.1093/toxsci/kfac067

**Published:** 2022-06-23

**Authors:** David Rider, Simon Chivers, Julia Aretz, Mona Eisermann, Kathrin Löffler, Judith Hauptmann, Eliot Morrison, Giles Campion

**Affiliations:** Silence Therapeutics GmbH, Berlin 13125, Germany; Integrated Biologix, Basel CH-4051, Switzerland; Silence Therapeutics GmbH, Berlin 13125, Germany; Silence Therapeutics GmbH, Berlin 13125, Germany; Silence Therapeutics GmbH, Berlin 13125, Germany; Silence Therapeutics GmbH, Berlin 13125, Germany; Silence Therapeutics GmbH, Berlin 13125, Germany; Silence Therapeutics PLC, London W14 8TH, UK

**Keywords:** GalNAc, lipoprotein (a), nonhuman primate, rat, siRNA

## Abstract

SLN360 is a liver-targeted N-acetyl galactosamine (GalNAc)-conjugated small interfering RNA (siRNA) with a promising profile for addressing lipoprotein (a)-related cardiovascular risk. Here, we describe the findings from key preclinical safety studies. *In vitro*, SLN360 specifically reduced *LPA* expression in primary human hepatocytes with no relevant off-target effects. In rats, 10 mg/kg subcutaneous SLN360 was distributed specifically to the liver and kidney (peak 126 or 246 mg/g tissue at 6 h, respectively), with <1% of peak liver levels observed in all other tested organs. *In vitro*, no genotoxicity and no effect on human Ether-a-go-go Related Gene currents or proinflammatory cytokine production was observed, whereas *in vivo*, no SLN360-specific antibodies were detected in rabbit serum. In rat and nonhuman primate 29-day toxicology studies, SLN360 was well tolerated at all doses. In both species, known GalNAc-conjugated siRNA-induced microscopic changes were observed in the kidney and liver, with small increases in alanine aminotransferase and alkaline phosphatase observed in the high dose rats. Findings were in line with previously described siRNA-GalNAc platform-related effects and all observations were reversible and considered nonadverse. In cynomolgus monkeys, liver *LPA* messenger RNA and serum lipoprotein (a) were significantly reduced at day 30 and after an 8-week recovery period. No dose-related changes in safety assessment endpoints were noted. No SLN360-induced cytokine production, complement activation, or micronucleus formation was observed *in vivo*. The toxicological profile of SLN360 presented here is restricted to known GalNAc siRNA effects and no other toxicity associated with SLN360 has been noted. The preclinical profile of SLN360 confirmed suitability for entry into clinical studies.

One attractive proposition for achieving long-term, but reversible, knockdown of a target gene and protein is using oligonucleotide therapeutics. These molecules have a unique potential to theoretically downregulate any RNA transcript through utilization of natural cellular mechanisms (Roberts *et al.*, 2020). Single-stranded antisense oligonucleotides (ASOs) were the forerunners in this field, achieving the earliest regulatory approvals (Roberts *et al.*, 2020). In the past 10 years, development of molecules in this class has grown rapidly fueled by the advancement in technologies for increased safety through specific cellular targeting mechanisms and the growth in the small interfering RNA (siRNA) field (Setten *et al.*, 2019). Early attempts using these mechanisms utilized unmodified or unconjugated oligonucleotide therapeutics, particularly ASOs, that were associated with numerous dose-limiting side effects including proinflammatory cytokine induction, thrombocytopenia and irreversible toxicity in the kidney and liver (Broering *et al.*, 2014; Chi *et al.*, 2017; Frazier, 2015; Henry *et al.*, 2016; Meng and Lu, 2017; Sewing *et al.*, 2017). Furthermore, the intrinsic properties of siRNA, eg, size and net negative charge, prevent generalized cellular uptake (Hu *et al.*, 2020), which hampered their early development.

Therefore, one of the most important recent developments in the oligonucleotide therapeutics field was the conjugation of ASOs and siRNA molecules to the N-acetyl galactosamine (GalNAc) moiety via novel linkers. The GalNAc residues bind to the asialoglycoprotein receptor (ASGPR) which is expressed almost exclusively on hepatocytes across species (ASGR1 Expression Profile, 2021; ASGR2 Expression Profile, 2021; Springer and Dowdy, 2018), targeting these conjugated oligonucleotides specifically to these cells and reducing inappropriate uptake by other tissues. In the case of GalNAc-conjugated siRNA molecules, this unique targeting of the liver and the mediation of a precise knockdown of a known target through Watson-Crick base-pairing has led to a generally predictable set of toxicological effects in early preclinical studies (Berman *et al.*, 2014, 2016; Henry *et al.*, 2016; Janas *et al.*, 2016, 2018). The exception to this can be seen through off-target toxicology, mediated through the predicted or unexpected knockdown of the expression of other genes, the use of novel chemical modifications to the bases and/or linker to the GalNAc moiety or exaggerated pharmacology.

Lipoprotein (a) [Lp(a)] was first reported in 1963 (Berg, 1963). Lp(a) is an LDL-like particle containing 2 proteins, apolipoprotein B-100 (apoB), encoded by the *APOB* gene, and apolipoprotein (a) [apo(a)], encoded by the *LPA* gene, that are covalently linked through a disulfide bridge. In the last 15 years, convincing evidence has been generated from large population-based observational studies that point to its causal role in cardiovascular disease (CVD; Kamstrup *et al.*, 2008; Erqou *et al.*, 2009). Strong relationships have been demonstrated between Lp(a) and the risk of cardiac arrest, stroke (Erqou *et al.*, 2009) and peripheral arterial disease (Gurdasani *et al.*, 2012). The pathophysiological mechanisms of Lp(a) in CVD are thought to be multifactorial and have been reviewed extensively elsewhere (Kostner *et al.*, 2018; Spence and Koschinsky, 2012; Tsimikas, 2017).

Current lipid-modifying agents are largely ineffective at reducing serum Lp(a) levels (van Capelleveen *et al.*, 2016), with monoclonal antibody PCSK9 inhibitors showing modest reductions (Korneva *et al.*, 2021; O’Donoghue *et al.*, 2019; Ray *et al.*, 2019). Several oligonucleotide therapeutics are already in clinical development (Swerdlow *et al.*, 2022), including SLN360 (Nissen *et al.*, 2022). SLN360 is a 19mer double-stranded GalNAc-conjugated siRNA targeting *LPA* messenger RNA (mRNA), the gene that encodes apo(a). Following binding and receptor-mediated cellular uptake by endocytosis, *LPA* mRNA is then targeted for degradation through RNA interference in the RNA-induced silencing complex via the antisense strand of the siRNA, thus reducing the production and release of Lp(a) by the liver (Rider *et al.*, 2022).

A toxicology safety evaluation program was conducted to support the clinical development of SLN360. The cynomolgus monkey was identified as a relevant species for toxicology testing of SLN360 because expression of *LPA* is restricted to humans, great apes and old-world monkeys (BLAST search performed on NM_005577.4 *Homo sapiens* lipoprotein(a) (*LPA*), mRNA), and thus provided the only readily available option to investigate target-specific toxicological effects of SLN360. Rat studies were performed to understand any potential liabilities that are unrelated to the on-target effects of the molecule, including any potential unidentified off targets that are relevant across species or those associated with chemical modifications within the molecule.

## MATERIALS AND METHODS

###  

####  

##### Animal welfare statement

All animal studies were performed in compliance with Institutional Guidelines following Directive 2010/63/EU and amendments on the approximation of laws, regulations, and administrative provisions of the Member States regarding the protection of animals used for experimental and other scientific purposes or in accordance with the requirements of the Animals (Scientific Procedures) Act 1986 and associated guidelines.

##### *In silico* identification of potential off-target genes

The design of the chosen siRNA sequence for SLN360 was initially guided *in silico* using proprietary software to optimize the efficacy of the knockdown on a sequence-based and thermodynamic level, as well as extensive *in silico* predictions of potential on targets and off targets in 4 primary species: *Homo sapiens* (human), *Macaca fascicularis* (cynomolgus), *Mus musculus* (mouse), and *Rattus norvegicus* (rat).

*In silico* off-target analysis was performed using proprietary sequence analysis software. Selected 19-mer oligonucleotides from the target mRNA were aligned with transcriptomic databases from NCBI and Ensembl (accessed October 18, 2019). Orthologous transcripts of the target gene from each of the 4 species were extracted from these databases. Significant alignments of target oligonucleotides with these orthologous transcripts are designated as “on targets,” whereas alignments with other transcripts are considered to be “off targets.”

Rigorous filtering was performed to eliminate sequences with significant predicted off targets. A key component of this filtering is the number of mismatches in the aligned predicted off target sequence. Predicted off-target sequences featuring perfect matches (ie, no mismatches) were filtered, as were those featuring only 1 mismatch. Predicted off-target sequences featuring 2 or 3 mismatches are considered to be of lower concern for off-target RNA binding and cleavage (Becker *et al.*, 2019). If a sequence had 2–5 unique genes with predicted off targets featuring 3 mismatches it was considered for further in-depth analysis; sequences with the number of predicted 3-mismatch off-target genes >2–5 were filtered out.

##### Hepatocyte treatment and mRNA analysis

Primary human hepatocytes (obtained from a single donor) and media were sourced from Life Technologies. As described by the manufacturer, primary hepatocytes were thawed and plated in Williams’ E medium (Life Technologies), supplemented with 5% fetal bovine serum, 1 µM Dexamethasone in dimethyl sulfoxide (DMSO) (final concentration of DMSO = 0.01%) and 3.6% v/v of Thawing/Plating Cocktail-A (Thermo Fisher Scientific, CM3000).

SLN360 was serially diluted in plating media to yield a final concentration range of 30 nM to 1 µM. Dharmacon control (antisense 5′-3′ CUUACUCUCGCCCAAGCGA, sense 5′-3′ UCGCUUGGGCGAGAGUAAG, for off-target screening) was diluted to yield a final concentration of 300 nM. Twenty five microliters of 5× solution of each concentration were distributed into 12 wells of collagen I-coated 96-well plates (Life Technologies) containing 50 µl of plating media. Immediately after this step, human primary hepatocytes in plating media were added to the siRNA solutions at a density of 35 000 cells per well. Final volume in each well was 125 µl. Plates were then incubated at 37°C in a 5% CO_2_ atmosphere for 24 h. Subsequently, cells were lysed, lysates were incubated and shaken (500 rpm) at room temperature for 30 min before being frozen at −80°C until RNA extraction as described below.

Total RNA was extracted using the InviTrap HTS 96-well kit (Stratec molecular GmbH, Berlin, Germany) according to the manufacturer’s instructions. Ten microliters of RNA-solution was used for gene expression analysis by reverse transcription-quantitative polymerase chain reaction (RT-qPCR) performed with an ABI StepOne Plus (Applied Biosystems, part of Thermo Fisher Scientific, Massachusetts) using standard protocols for RT-qPCR (48°C 30 min, 95°C 10 min, 40 cycles at 95°C 15 s followed by 60°C 1 min). Analyses for *LPA* and all identified potential off-target genes were performed in single plex assays (primers: 300 nM, probe: 100 nM). *APOB* (200 nM) and *ACTB* (beta actin) (300 nM) were run in a multiplex assay adding 100 nM of each probe to the standard mixture. Details of primer sequences are shown in Supplementary Table 1.

##### Rat biodistribution

The biodistribution of SLN360 was explored in male and female Wistar rats (4 M and 4 F per timepoint), following a single 10 mg/kg subcutaneous (s.c.) injection. SLN360 concentration was determined in plasma, blood cell pellet, whole blood, liver, kidney, lungs, spleen, heart, bone marrow, testes, ovary, uterus, and thymus over 21 days following dosing using an anion exchange high-performance liquid chromatography (AEX-HPLC) assay.

##### AEX-HPLC in biodistribution

The method is based on the specific hybridization of the SLN360 antisense strand with a 15-mer complementary PNA strand in a 1:1 stoichiometry. The PNA is labeled with an Atto425 fluorescence dye and yields a specific signal in the subsequent analysis by AEX-HPLC coupled to a fluorescence detector. To avoid the formation of PNA/RNA triplex structures directly after the hybridization process, a deoxyribonucleic acid (DNA) blocking oligonucleotide is added. The DNA blocking oligonucleotide forms a stable duplex with excess PNA at room temperature. At HPLC column temperature the PNA/DNA duplex dissociates and the excess fluorescently labeled PNA elutes at the beginning of the HPLC gradient without interfering with analyte analysis. The method was developed without the need for any analyte extraction from plasma or tissue; so that a recovery of 100% is maintained over the process (no internal standard is needed). Quantification is performed using an external calibration curve generated after spiking SLN360 at defined concentrations into blank plasma or tissue matrix and was used in the non-GLP (good laboratory practice) biodistribution study.

##### Liquid chromatography-tandem mass spectrometry in GLP safety studies

For the second study, the concentration of SLN360 in plasma, liver, and kidney was determined via liquid chromatography-tandem mass spectrometry. An antisense strand with 13C and 15N modifications on the 2′-O-methyl-cytosine nucleosides yielding a 48 Da mass shift was used as internal standard. Plasma samples were prepared via phenol chloroform extraction before injection. An Acquity ultra performance liquid chromatography I-Class equipped with an Acquity BEH C18, 1.7 µm 50 × 2.1 mm column from Waters was used. Separation was performed via ion-paring reverse-phase chromatography with ethylenediaminetetraacetic acid, triethylamine, and hexafluorisopropanol as mobile phase modifiers in water and methanol at 80°C. Detection was performed with a Sciex API 6500+ in negative mode following the 769.4→94.9 and 775→94.9 transition for SLN360 and the internal standard, respectively.

##### Genotoxicity studies

SLN360 was tested *in vitro* (mutagenicity potential in a bacterial AMES test, clastogenicity and aneugenic potential in mouse lymphoma cells containing the *tk* locus and human lymphocytes) and *in vivo* (rat bone marrow micronucleus test) in prescribed genotoxicity studies for clinical candidates. The studies were performed in line with ICH guidelines provided for these studies and details of the methods can be found in the Supplementary Methods.

##### Immune activation studies

The potential immune interactions of SLN360 were assessed *in vitro* in cynomolgus and human whole blood. SLN360 was added to whole blood of either species (*n* = 4 per species) at a concentration of 10 µM and cytokine production (interleukin [IL]-1β, IL 2, IL-6, IL-8, IL-10, IL-13, tumor necrosis factor [TNF]-α, interferon [IFN]α, and IFNγ) was measured at 0, 2, 6, or 24 h using a multiplex assay (Bio-Plex 200 Luminex system). Detailed methods can be found in the Supplementary Material.

##### Potential antidrug-antibody production

SLN360 was coupled to a carrier protein (Limulus polyphemus hemolymph hemocyanin), formulated in adjuvant, and injected intramuscularly on 6 separate occasions to 3 female Zimmerman rabbits (1 mg/kg [dose 1], 0.5 mg/kg [doses 2–5], and 0.25 mg/kg [dose 6]) over a period of 16 weeks. Detailed methods can be found in the Supplementary Material.

##### Rat 29-day GLP toxicology study

Sprague Dawley rats received 5 once weekly s.c. injections (dosing on days 1, 8, 15, 22, and 29) of 0.9% saline control or SLN360 (10 M/10 F per group, 3, 10, or 30 mg/kg) at a dose volume of 10 ml/kg followed by an 8-week recovery period (3 M/3 F in saline and top dose group). An *in vivo* micronucleus test was included in the study with a positive control group administered 20 mg/kg Cyclophosphamide (10 M/10 F) as a single dose via oral gavage approximately 24 h prior to necropsy (10 ml/kg). Assessment of toxicity was based on mortality; clinical observations; body weights; food consumption; ophthalmic observations; modified Irwin observations; clinical and anatomic pathology; and micronucleus assessment. Blood samples were collected for toxicokinetic (TK) evaluation, and liver and kidney samples were collected for non-GLP bioanalysis.

##### Cynomolgus 29-day GLP toxicology study

Cynomolgus monkeys received 5 once weekly s.c. injections (dosing on days 1, 8, 15, 22, and 29) of saline control or SLN360 (3 M/3 F per group, 30, 100, or 150/200 mg/kg) at a dose volume of 0.75–1.0 ml/kg, followed by an 8-week recovery period (2 M/2 F in saline and top dose group). Males dosed at 150 mg/kg on days 1–15 and 200 mg/kg on days 22 and 29. Females dosed at 150 mg/kg on day 1 and 200 mg/kg days 8–29. Clinical chemistry, hematology, circulatory, and ECG parameters (jacketed telemetry), respiratory rate, neurobehavior, plasma cytokines (IL-1β, IL-2, IL-6, IL-8, IL-10, IFNγ, and TNF-α), complement activation (C3a, Bb, and sC5b-9) and CRP levels were measured prior to the first dose, at parameter-specific timepoints during the study and at the end of the recovery period. Liver *LPA*, *APOB*, and plasminogen (*PLG*) mRNA levels were measured at necropsy on day 30 and following the recovery period.

##### Histology

Tissues collected from the GLP toxicology studies were preserved in 10% neutral buffered formalin, unless otherwise indicated (Supplementary Table 2). Tissues were then embedded in paraffin wax, sectioned at a nominal 5-µm thickness and stained with hematoxylin and eosin using standard techniques. Scanned slides were analyzed and images captured using Aperio Imagescope software v12.1.

##### Liver mRNA analysis

Approximately 10 mg of liver tissue from each animal was disrupted and homogenized in a Mixer Mill MM 400 (Retsch GmbH, Haan, Germany) using 3 mm tungsten carbide beads (Qiagen, Hilden, Germany) and total RNA was prepared with RNeasy Fibrous Tissue Mini Kit (QIAGEN, Venlo, The Netherlands) according to the manufacturer’s instructions.

RNA integrity was confirmed using a 2100 Bioanalyzer (Agilent Technologies, Inc., Santa Clara, California). One hundred nanograms per reaction of total RNA was used for RT-qPCR performed with a QuantStudio 6 Flex (Applied Biosystems part of Thermo Fisher Scientific, Massachusetts) using Takyon One-Step Low Rox Probe 5X MasterMix dTTP (Eurogentec, Seraing, Belgium) and a fast protocol (48°C 10 min, 95°C 3 min, 40 cycles at 95°C 3 s followed by 60°C 20 s) in a 384-well format. All reactions were performed using a single plex approach with primers at a concentration of 300 and 100 nM for the probe. RT-qPCR analysis was performed in triplicate for each sample.

The amount of *LPA*, *APOB*, and *PLG* mRNA normalized to the endogenous reference *ACTB* relative to the average of the levels in control animals was calculated using the formula: Fold-change = 2^−^^ΔΔ^^*C*^^t^. Statistical analysis was performed comparing the control group to the relevant dose groups using a 1-way ANOVA with Dunnett’s post hoc analysis. Differences were considered significant if the *p* value was < .05. All analyses were performed using Graphpad Prism Software version 8.4.3.

##### Serum Lp(a) ELISA

Serum Lp(a) from cynomolgus monkey was measured by ELISA according to the manufacturer‘s instructions [Human Lp(a) ELISA, Mercodia, Sweden]. Data from each animal were converted to nmol/l using the following calculation: multiplication of U/L by 0.1254 to give mg/dl, followed by multiplication of the mg/dl by 2.4.

Serum samples were taken 2 or 3 times prior to commencement of dosing and used to generate the baseline (100%) Lp(a) level for each individual animal. The percentage change from the baseline level of Lp(a) was then calculated for each timepoint for each individual animal using the following equation:

% Baseline Lp(a)=((Lp(a) level)/(average Lp(a) baseline))* 100%

## RESULTS

###  

#### SLN360 Specifically Downregulates *LPA* in Primary Hepatocytes

**Figure 1.**
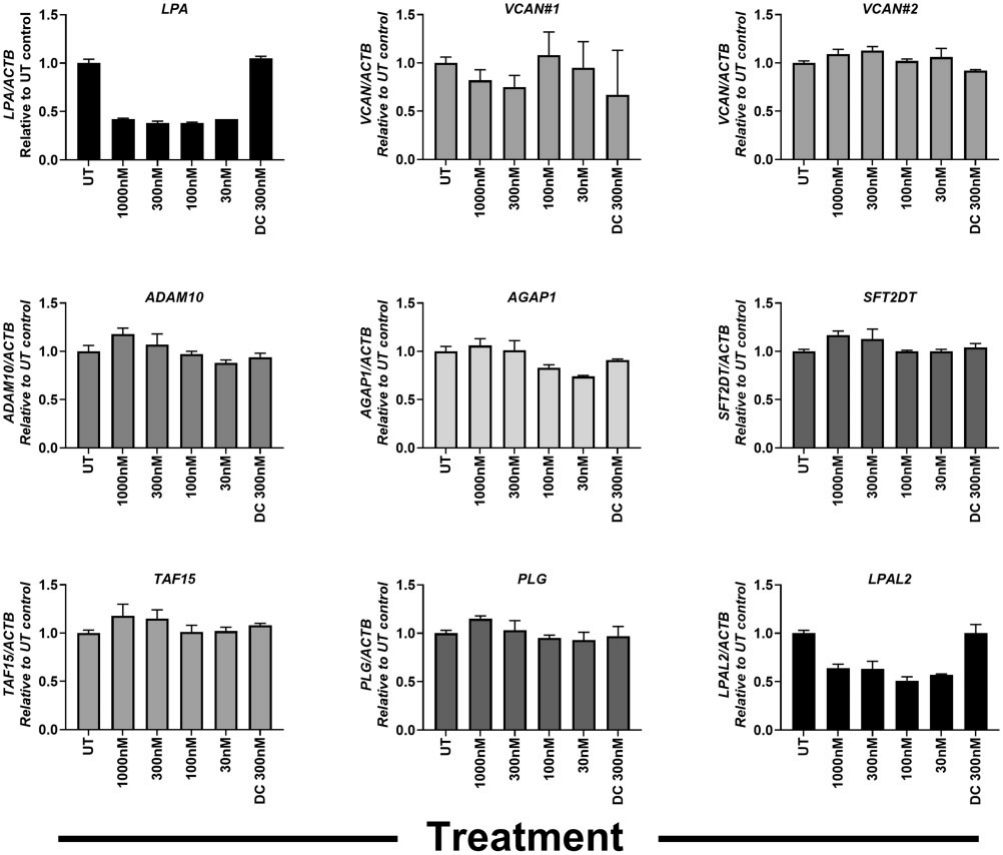
Effect of SLN360 on downregulation of predicted human off targets. Primary human hepatocytes were incubated with SLN360 (30–1000 nM) for 24 h before extraction of mRNA and analysis of effects on the indicated potential off-targets. Data are presented as the mean ± SD from 3 individual experiments (3 replicates were contained in each individual experiment). Abbreviations: *ADAM10*, disintegrin and metalloproteinase domain-containing protein 10; *AGAP1*, Arf-GAP with GTPase, ANK repeat and PH domain-containing protein 1; DC, dharmacon control sequence; *LPA*, lipoprotein (a); *LPAL2*, lipoprotein(a)-like 2, pseudogene; *PLG*, plasminogen; *SFT2DT*, vesicle transport protein SFT2B; *TAF15*, TATA-box binding protein-associated factor 15; *VCAN*, versican core protein.

As part of the selection of SLN360, the siRNA sequence was designed and evaluated using proprietary software, including extensive predicted on- and off-target analyses. In human, 7 potential off-target genes were identified in silico, 6 of which are expressed in the liver and showed very little cross-species overlap (Supplementary Table 3). The majority of these 6 liver-expressed genes feature ≥3 mismatches, suggesting a low likelihood of true off-target RNA binding and cleavage. The one predicted off target with no mismatches is a noncoding RNA from the pseudogene *LPAL2* [lipoprotein(a)-like 2], again indicating low concern for off-target effects. Furthermore, there were no identified seed similarities with known microRNAs in any species. To confirm these in silico results, the 6 liver-expressed predicted off-target genes were validated *in*  *vitro*. Additionally, the effect on the closely related *PLG* gene was also investigated. We have previously demonstrated that in cynomolgus hepatocytes, SLN360 specifically and potently downregulates *LPA* (IC_50_ 0.1 nM), with no effects on the expression of *APOB* or *PLG* mRNA (Rider *et al.*, 2022). Here, primary human hepatocytes were exposed to SLN360 at concentrations up to 1000 nM. As expected, *LPA* mRNA was reduced by approximately 60% at all concentrations tested. Except for the pseudogene *LPAL2*, none of the other identified potential off targets, nor *PLG*, showed any concentration-dependent reduction in mRNA expression up to SLN360 concentrations of 1000 nM, over 300-fold the IC_50_ for SLN360 in primary human hepatocytes (Figure 1). These results indicate that SLN360 is specific and does not affect any of the potential off targets identified during *in*  *silico* screening.

#### SLN360 Plasma Exposure

The pharmacokinetic and toxicokinetic properties of SLN360 in rats and the toxicokinetic properties in non-human primates (NHP) were evaluated (Table 1). The pharmacokinetic properties of NHP have been reported elsewhere (Rider *et al.*, 2022). In rats, a single 10 mg/kg dose resulted in maximum plasma levels being observed in males (3739 ng/ml) and females (4412 ng/ml) at the first measurement point (1 h), with whole blood concentration approximately half the plasma concentration at the same time point (1815 and 2248 ng/ml in males and females, respectively). A negligible quantity of SLN360 (93 ng/g) was found in the cell pellet after 1 h and was undetectable 6 h after dosing, suggesting that blood cell binding and uptake of SLN360 was limited.

**Table 1.** Pharmacokinetic and Toxicokinetic Properties of SLN360

Day	Dose Group	Nominal Dose (mg/kg/week)	Actual Dose^*a*^ (mg/kg/week)	Sex (*n*)^*b*^	*C*_max_ (ng/ml)	*T*_max_ (h)	AUC_0–48_ (h*ng/ml)	*t*_1/2_ (h)
Rat biodistribution pharmacokinetics								
1		10		M (4)	3739	1	7109^*c*^	2.26
				F (4)	4412	1	6316^*c*^	1.92
Rat 29-day toxicokinetics								
1	2	3		M (3)	452	1	1550	0.89
				F (3)	518	0.33	1440	0.63
	3	10		M (3)	2080	2	9220	NC^*d*^
				F (3)	2700	0.33	8240	0.80
	4	30		M (3)	14 400	1	47 100	0.79
				F (3)	13 400	1	38 300	0.68
29	2	3		M (3)	529	1	1750	1.15
				F (3)	620	1	1550	0.69
	3	10		M (3)	4840	2	24 400	NR^*e*^
				F (3)	3380	1	8300	0.87
	4	30		M (3)	18 600	1	121 000	1.90
				F (3)	15 900	1	59 800	0.89
NHP 29 day toxicokinetics^*f*^								
1	2	30	24	M (3)	10 100	2	108 000	2.48
			24	F (3)	7740	2	77 600	4.33
	3	100	82	M (3)	19 900	4	293 000	5.49
			82	F (3)	21 400	4	312 000	4.84
	4	150	124	M (5)	34 300	4	533 000	4.65
			124	F (5)	44 900	4	572 000	4.76
29	2	30	30	M (3)	12 500	2	120 000	3.24
			24	F (3)	7260	4	83 100	NR
	3	100	100	M (3)	27 000	4	396 000	NR
			84	F (3)	25 400	4	325 000	NR
	4	200	200	M (5)	64 900	6	938 000	NR
			166	F (5)	50 100	6	781 000	NR

a Formulation errors led to mis-dosing on TK days 1 for all animals and day 29 females in NHP study.

b *n* indicates animals per timepoint.

c Data for this study are AUC_0–24_ as final PK timepoint was 24 h.

d NC, not calculated due to the inability to appropriately define an elimination phase or less than 3 sample timepoints used for determination of AUC_0–inf_ as to exclude *C*_max_.

e NR, not reported due to a lack of a distinct elimination phase.

f Dose level increased mid-study due to lack of evident toxicity.

The toxicokinetic properties of SLN360 were assessed in the 29-day GLP multiple dose toxicology studies. In the rat, plasma exposure (SLN360 *C*_max_ and area under the curve [AUC]_0__–__48_) increased with increasing dose from 3 to 30 mg/kg, but was greater than dose-proportional, indicating saturable ASGPR hepatic uptake and clearance process (most evident for males on day 29). For female animals, SLN360 *C*_max_ and AUC_0__–__48_ values were similar on days 1 and 29, indicating no accumulation of SLN360 in plasma after multiple doses of SLN360 in female rats. However, in males, *C*_max_ and AUC_0__–__48_ values were generally higher on day 29 than on day 1, indicating some accumulation of SLN360 after multiple doses of SLN360 in rats (most marked at 10 and 30 mg/kg). In the 3 and 10 mg/kg dose groups, after reaching *C*_max_, SLN360 concentrations declined rapidly, with mean *t*_1/2_ values ranging from 0.789 to 0.891 h on day 1 in males and from 0.633 to 0.804 h on day 1 in females. On day 29, *t*_1/2_ values ranged from 1.15 to 1.90 h, in males and from 0.687 to 0.887 h, in females. Sex differences in SLN360 exposure (*C*_max_ and AUC_0__–__48_) in the rat were more evident on day 29, compared with day 1 (approximately 2-fold or greater at 10 or 30 mg/kg, with higher exposure in males).

In NHP exposure, as assessed by mean SLN360 DN *C*_max_ and DN AUC_0__–__20_ values, increased with each increase in nominal dose level between 30 and 150 or 200 mg/kg/week. The observed increases in exposure were dose-proportional to the increases in actual dose level. No accumulation of SLN360 in plasma was observed after 5 once weekly s.c. doses in monkeys. *C*_max_ was achieved within 2–6 h following s.c. dosing, followed by rapid clearance with individual half-life values ranging between 2.42 and 5.88 h.

In summary, SLN360 displayed no marked blood cell uptake or binding with a typically short plasma half-life (NHP longer than rat) that is characteristic of GalNAc-siRNA molecules. Approximately 2-fold differences in plasma exposure were observed in males at higher doses in rats, but this was not observed in NHPs.

#### SLN360 Tissue Distribution Is Largely Restricted to Kidney and Liver

**Figure 2.**
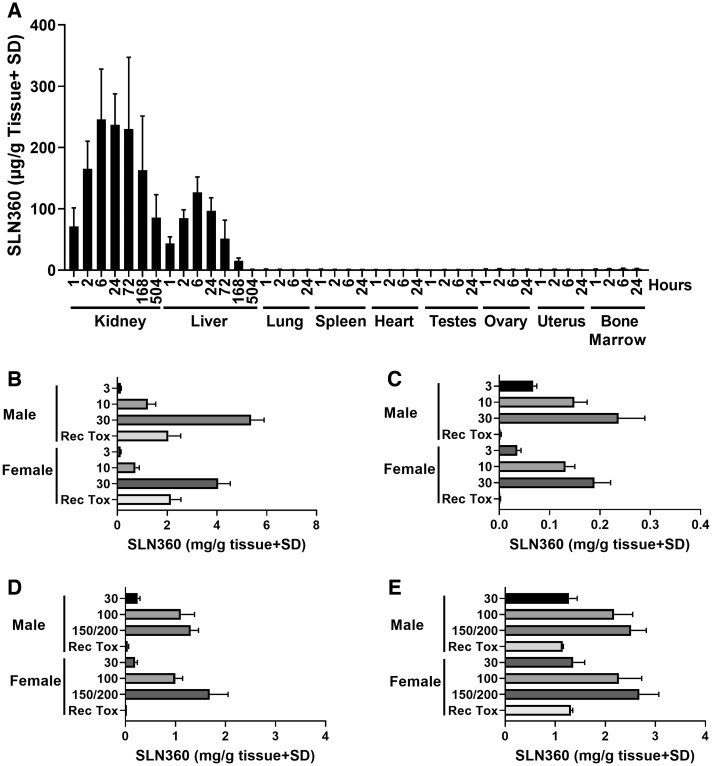
Distribution of SLN360 is restricted to kidney and liver. A, Distribution of SLN360 in various tissues in rats following a single s.c. dose of 10 mg/kg. Each column represents the mean concentration ± SD observed in 8 animals (4 F and 4 M) at each timepoint, with the exception of testes (4 M), ovary and uterus (4 F). B, Kidney and C, liver concentrations in rats following 5 weekly doses of SLN360 in a GLP toxicology study at the indicated doses (mg/kg). Data represent the mean concentration ± SD from 10 animals per dose/per sex. Recovery data are derived from 5 animals per sex. D, Kidney and E, liver concentrations in NHP following 5 weekly doses of SLN360 in a GLP toxicology study at the indicated doses (mg/kg). Data represent the mean concentration ± SD from 3 animals per dose/per sex. Recovery data are derived from 2 animals per sex. Abbreviation: s.c., subcutaneous.

The biodistribution profile of SLN360 was investigated in SD rats following a single 10 mg/kg s.c. injection. SLN360 content was determined in the indicated tissues over 21 days following dosing using an AEX-HPLC assay. Liver and kidney were the only organs with appreciable levels of SLN360 with comparable levels in all tissues in males and females (Figure 2A). Kidney exposure was approximately 2-fold that of the liver in males and females peaking at around 6 h postdose (mean levels 250 µg/g for males and 242 µg/g for females), before declining after 72 h. A long tissue half-life was evident for the kidney (approximately 265 h), with 35% of the peak level remaining after 21 days. Liver exposure peaked at 6 h (mean levels 128 µg/g for males and 126 µg/g for females), already declining at the next sampling time point (24 h) in both sexes. The other organs (lung, spleen, heart, testes, ovary, uterus, and bone marrow) had very low levels, generally not exceeding 1.5% of the peak level observed in kidney or liver, indicating limited exposure of these organs to SLN360. These observations are consistent with the specificity of the GalNAc hepatic delivery system and the main renal route of elimination (Janas *et al.*, 2018). The overall tissue half-life for the liver was moderate (approximately 50 h), with 12% of the peak level remaining after 7 days and only 1% after 21 days.

Additionally, the distribution of SLN360 into the kidneys and liver was assessed in 29-day GLP toxicology studies (Figs. 2B–E). In rats, SLN360 levels in the kidney were approximately increased compared with those found in the liver in a dose-dependent manner. At 3 mg/kg, the difference was 2-fold (male) or 3.7-fold (female) at day 30, whereas at 10 mg/kg it was 8.2-fold (male) and 5.7-fold (female). At the highest dose of 30 mg/kg, kidney levels were 22-fold the level found in the liver in both sexes, a disparity that was maintained and even enhanced through the recovery period. In contrast, levels of SLN360 in the NHP were approximately 2-fold higher in liver than kidney at termination at day 30 at the 2 highest doses in both sexes. At 30 mg/kg, there was a 5- to 7-fold enhancement in liver to kidney levels at day 30. Following recovery, liver levels were approximately 25-fold higher in males (1.15 mg/g compared with 0.05 mg/g) and 43-fold higher in females than levels in the kidney (1.32 mg/g compared with 0.03 mg/g).

In summary, SLN360 was detected at significant tissue concentrations in liver and kidney, whereas levels in other organs were extremely limited in the rat biodistribution study. A disparity in the distribution to kidney and liver was evident between rats and NHP, whereby SLN360 was preferentially found in the kidney of rats compared with the liver, whereas the opposite was observed in the NHP.

#### Toxicological Assessments

SLN360 underwent a series of toxicological assessments required by regulatory authorities for clinical entry (Table 2). SLN360 was shown to be nongenotoxic in a battery of prescribed *in vitro* and *in vivo* assessments and had no effect on human Ether-a-go-go Related Gene (hERG) currents *in vitro*. Cytokine induction was not induced in human or NHP whole blood *in vitro*, there was an absence of cytokine induction or complement activation in NHP *in vivo* and no antidrug antibodies could be detected in rabbits, suggesting SLN360 has low immunogenic potential.

**Table 2.** Safety Assessments Performed With SLN360

Study Type	Test System^*a*^	Treatment/Follow-Up Duration	Dose/Concentration	Findings
CV safety	*In vitro* hERG	*In vitro*	20 µg/ml	hERG tail currents were inhibited by <10% (99.02±2.05% relative tail current, [mean±SEM] of *n*=3 cells) by 20 µg/ml SLN360
Immunogenicity				
*In vitro* cytokine response	Whole blood (human and cynomolgus; *n* = 4 per species)	*In vitro*, 0, 2, 6, and 24 h	10 µM	No induction of IL-1β, IL-2, IL-6, IL-8, IL-10, IL-13, TNF-α, IFNα, and IFNγ by SLN360 was detected
* In vivo* antigenicity	Zimmerman Rabbits (3F)	112 days	1.0 mg (day 1) 0.5 mg (days 7, 14, 56, and 70) 0.25 mg (day 98)	No generation of SLN360-specific antibodies was detected
Genotoxicity				
AMES^*b*^	*Salmonella typhimurium*	*In vitro*	≤5 mg/plate	SLN360 did not induce mutations in the 5 strains tested
* In vitro* mammalian cell gene mutation assay^*b*^	Mouse lymphoma L5178Y (*tk*^+/−^) cells	*In vitro*	≤500 µg/ml	SLN360 did not induce mutation at the *tk* locus of mouse lymphoma L5178Y cells
* In vitro* micronucleus test^*b*^	Cultured human peripheral blood lymphocytes	*In vitro*	≤500 µg/ml	SLN360 did not induce micronuclei in cultured human peripheral blood lymphocytes
* In vivo* bone marrow micronucleus test	Rat; Sprague Dawley (5M/5F/group)	SLN360 29 days CPA	0, 3, 10, and 30 mg/kg, s.c., q7d 20 mg/kg, p.o., d29	SLN360 did not induce micronucleus formation following weekly dosing up to 30 mg/kg in the rat

*Abbreviations:* CPA, cyclophosphamide; s.c., subcutaneous.

a Detailed methods can be found in the Supplementary Material.

b All *in vitro* genotoxicity tests were carried out in the absence or presence of a rat liver metabolic activation system (S-9) fraction, as described in the Supplementary Material.

#### SLN360 Is Well Tolerated in 29-Day GLP Toxicology Studies

SLN360 was assessed for suitability to enter the clinic in 2 separate species. Cynomolgus monkeys were considered the most relevant preclinical species as the *LPA* target gene is only expressed in NHP and humans, whereas SD rats were used to understand any chemical structure-related toxicology only.

**Figure 3.**
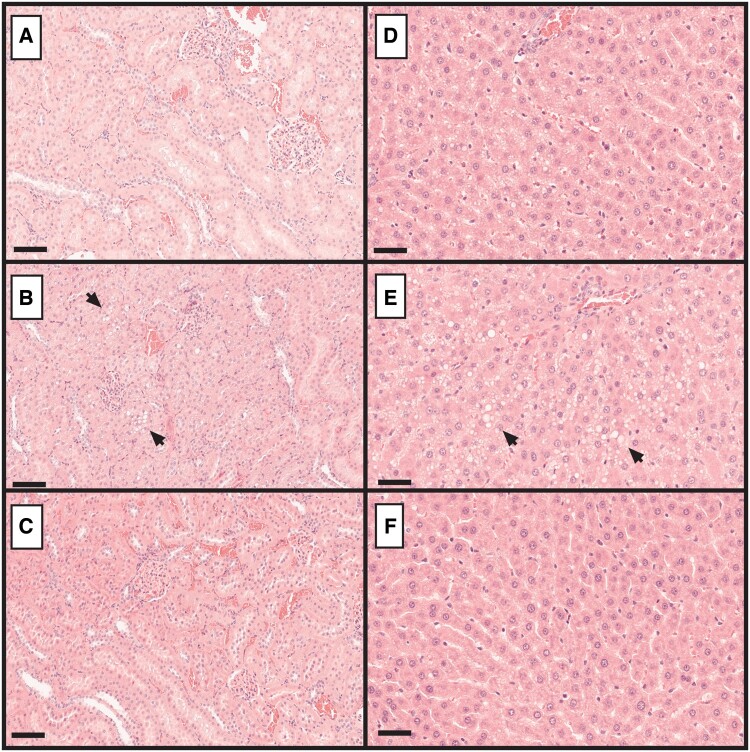
Histological observations in the rat kidney and liver. Representative tissue sections from kidney (A–C) and liver (D–F) stained with hematoxylin and eosin. A and D, control; B and E, high dose; and C and F, recovery animals. In kidney, tubular vacuolation is evident in the high dose group (B) indicated by arrows, but absent in the control (A) and recovery (C) animals. In liver, compared with the control (D) hepatocellular vacuolation is increased in the high dose group (E) example areas indicated by arrows and resolves after recovery (F). Images (A–C) at ×20 magnification and bar indicates 100 µm; (D–F) at ×40 magnification and bar indicates 50 µm.

Once-weekly s.c. administration of 3, 10, or 30 mg/kg SLN360 to male and female Sprague Dawley rats for 29 days (a total of 5 doses) was well tolerated, with no test article-related mortality; clinical, dose site, postdose, or ophthalmic observations; or effects on urinalysis, micronucleus induction, or organ weights. Reduced body weight gain during the dosing and recovery phases for females administered 30 mg/kg was slight in magnitude. Reversible tubular vacuolation (severity grade 1) was observed in the kidney tubules of both sexes administered 30 mg/kg correlated with clinical pathology changes (approximately 1.2-fold increased creatinine and urea concentrations) but was considered nonadverse (Figs. 3A and 3B). Hepatocyte vacuolation was increased in the liver of males at all doses and females administered ≥10 mg/kg compared with control animals (Figs. 3D and 3E). This was associated with moderate increases in alanine transaminase (ALT) activity (2-fold, males) and alkaline phosphatase (ALP) at doses ≥10 mg/kg in males (2-fold) and 30 mg/kg in females (1.5-fold). No changes in aspartate aminotransferase (AST), gamma-glutamyl transferase (GGT) or total bilirubin were noted (Supplementary Figure 1 and Supplementary Table 4). All microscopic and liver enzyme changes were no longer evident following recovery (Figs. 3C and 3F). Additionally, no salient microscopic changes were noted in any of the other organs examined (see Supplementary Table 2 for full-list). The NOAEL from the rat study was considered to be 30 mg/kg.

**Figure 4.**
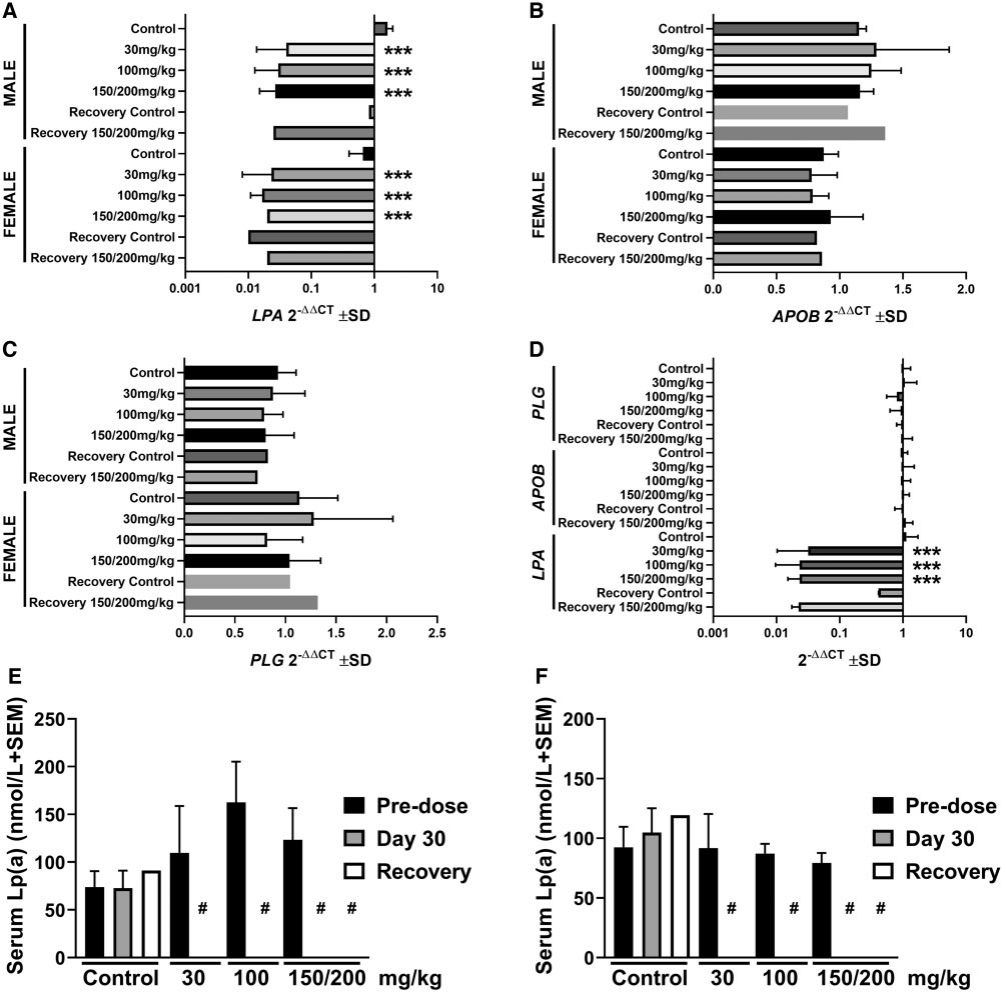
SLN360 specifically reduces *LPA* mRNA and serum Lp(a). Liver mRNA levels of *LPA* (A), *APOB* (B), and *PLG* (C) displayed by sex and all animals combined (D). Data represent the mean ± SD of 3 animals per sex (all dose groups) or mean of 2 animals per sex (recovery groups). ***p < .001 as determined using a 1-way ANOVA with Dunnett’s post hoc analysis. No significant differences were observed between control and dose group *APOB* and *PLG* levels. Serum lipoprotein (a) [Lp(a)] in male (E) and female (F) animals. Data represent the mean ± SEM of 5 animals per sex (control and high dose on predose and day 30), 2 animals per sex (recovery timepoint) and 3 animals for 30 and 100 mg/kg. #below limit of detection of the assay, therefore no statistical analysis was applied. Abbreviations: *LPA*, apolipoprotein(a); *APOB*, apolipoprotein B-100; *PLG*, plasminogen.

Once-weekly s.c. administration of 30, 100, or 150/200 mg/kg SLN360 to male and female cynomolgus monkeys for 29 days (a total of 5 doses) was well tolerated with no SLN360-related mortality, clinical, postdose, ophthalmic, urinalysis, or neurological observations. There were no SLN360-related effects on body weight; food consumption, hematology, urinalysis, safety pharmacology endpoints (blood pressure, respiration rate, heart rate or ECGs [qualitative and quantitative]). No significant postdose SLN360-related local effects were seen, except for a single erythema in 1 male in the 100 and 150/200 mg/kg NHP groups. No evidence of complement activation or enhanced cytokine production in relation to SLN360 was observed. As expected, SLN360 induced specific near total reduction of *LPA* mRNA and serum Lp(a) at day 30 with no return to normality observed following the 8-week recovery period (Figure 4), with equivalent effects observed in female and male animals. This observation is in line with the high concentrations at the end of the recovery period and the long half-life of SLN360 in the liver (Figure 2E).

Group mean liver weights and ratios were higher for animals administered 100 or 150/200 mg/kg, compared with concurrent controls, correlating with the macroscopic, pale appearance and/or enlargement of the liver at 100 or 150/200 mg/kg.

**Figure 5.**
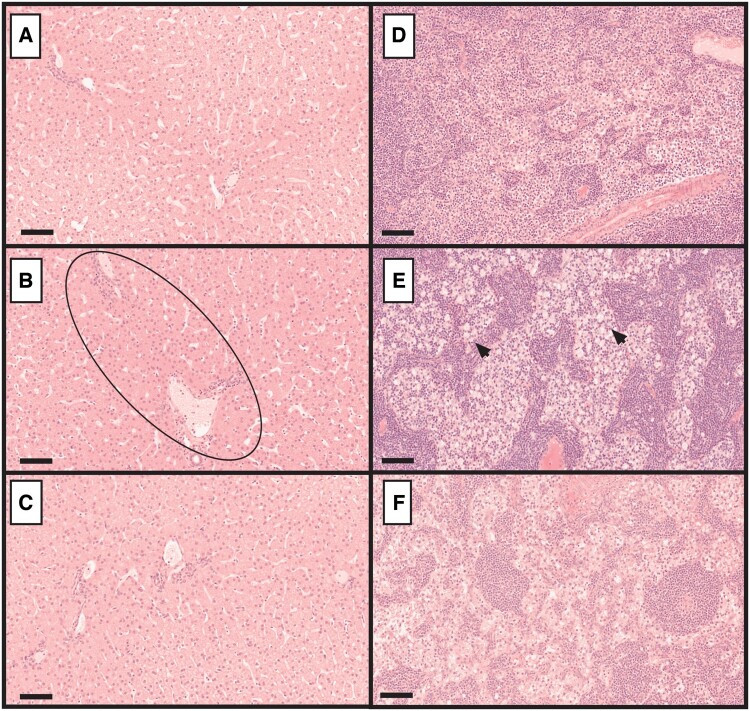
Histological observations in the cynomolgus liver and lymph nodes. Representative tissue sections from liver (A–C) and mesenteric lymph node (D–F) stained with hematoxylin and eosin. A and D, control; B and E, high dose; and C and F, recovery animals. In liver, hepatocellular hypertrophy is evident in the high dose group (B) indicated by ellipse, but absent in the control (A) and recovery (C) animals. In mesenteric lymph nodes, vacuolated macrophages are evident in the high dose group (E) indicated by arrows compared with control animals (D) and there is a partial resolution following recovery (F). Images at ×20 magnification and bar indicates 100 µm.

Microscopically, SLN360-related findings were recorded for the liver and examined lymph nodes (Figure 5). In the liver, diffuse hepatocyte hypertrophy was recorded for all males administered 150/200 mg/kg (minimal to slight) and 4 females administered 100 or 150/200 mg/kg (minimal; Figs. 5A and 5B). These changes were not associated with increases in ALP, ALT, AST, GGT, or bilirubin (Supplementary Figure 2 and Supplementary Table 5). In the mesenteric, mandibular, and/or axillary lymph nodes, nonadverse reversible vacuolated macrophages (minimal to slight) were observed in most animals administered 30, 100, or 150/200 (Figs. 5C–F).

In conclusion, once weekly s.c. administration of up to 200 mg/kg SLN360 to cynomolgus monkeys on 5 occasions over 29 days was well tolerated. Reversible findings in the liver (increased weight with diffuse hepatocyte hypertrophy) and reversible lymph node changes (vacuolated macrophages), were considered nonadverse. The NOAEL was 200 mg/kg.

## DISCUSSION

The nonclinical toxicology program conducted to-date with SLN360 includes repeat-dose toxicity studies in 2 species (rat and cynomolgus monkey), with integrated safety pharmacology core battery assessment, genotoxicity studies, and an *in vitro* cytokine production study using cynomolgus monkey and human whole blood. These studies were conducted to determine the suitability of the molecule to enter clinical studies.

SLN360 was subjected to tests for mutagenicity as well as clastogenicity and aneugenic potential in bacterial and mammalian assays systems *in vitro* as well as rat bone marrow micronucleus formation *in vivo*. These experiments confirmed the lack of genotoxicity of SLN360 *in vitro* and *in vivo*. This is fully aligned with the general lack of genotoxicity seen with this class of siRNAs (Berman *et al.*, 2016; Janas *et al.*, 2016).

All clinically tested ASOs and siRNAs have varying degrees of phosphorothioate (PS) replacing one or several phosphodiester internucleotide linkages in the oligonucleotide backbone. Therapeutic ASOs are often fully modified with PS internucleotide linkages, while the double-stranded nature of siRNA appears to reduce the need for full-PS modification. A number of hybridization-independent toxicities of oligonucleotide therapeutics, particularly of ASOs, are thought to be in part attributable to the PS modification, such as inflammatory cytokine release, thrombocytopenia, and irreversible liver and kidney toxicity (Chi *et al.*, 2017; Frazier, 2015; Henry *et al.*, 2016; Sewing *et al.*, 2017). No thrombocytopenia, adverse kidney or liver toxicity was observed with SLN360 in the studies presented here, which is in line with clinical findings for similar approved siRNA molecules, such as patisiran and inclisiran (Balwani *et al.*, 2020; Garrelfs *et al.*, 2021; Ray *et al.*, 2020). Inflammatory cytokine release has also been attributed to the use of chemically unmodified bases in their structure (Meng and Lu, 2017). Incorporation of the 2ʹ-O-methyl ribosugar and the 2ʹ-deoxy-2ʹ-fluoro sugar modification has been shown to mitigate this (Broering *et al.*, 2014). These modifications are generally used for siRNA therapeutics and similar agents have been tested in animals and humans with little evidence of safety or tolerability concerns (Janas *et al.*, 2019). SLN360 incorporates these bases into its structure and no inflammatory cytokine release or complement activation *in vitro* or *in vivo* has been detected.

Cardiovascular safety through initial *in vitro* hERG testing is a prerequisite for many development candidates that are deemed to be chemically manufactured (ICH Topic S7B: Guidance for Industry: Nonclinical Evaluation of the Potential for Delayed Ventricular Repolarization (QT Interval Prolongation) by Human Pharmaceuticals, 2005). GalNAc siRNA currently fall into this category and SLN360 therefore underwent hERG testing. SLN360 had no effect *in vitro* on hERG currents and *in vivo* had no effect on ECG or other telemetry measurements. These data are typical for oligonucleotide therapeutics and support the position that the *in vitro* hERG assay does not provide any specific value and is not warranted due to the intrinsic properties of GalNAc siRNA (limited systemic exposure, specific receptor-mediated uptake, unlikely intracellular access), with an *in vivo* ECG analysis preferred (Berman *et al.*, 2014).

The observed toxicity with SLN360 in the 29-day GLP toxicology studies is in line with previously published findings for GalNAc siRNAs (Janas *et al.*, 2018). Studies in the NHP revealed adaptive changes in the draining lymph nodes and liver. These changes are generally observed with this class of molecules and are independent of the sequence-directed target specificity of these drug candidates. Findings in the rat are characterized by changes in the liver and kidney. These are considered related to intracellular accumulation of SLN360 uptake in the liver and kidney (clearance route), and are aligned with the demonstrated preferential distribution of SLN360 to the liver and kidney and the intrinsic properties of GalNAc-conjugated siRNA, which prevent generalized cellular uptake (Hu *et al.*, 2020).

The vacuolation/hypertrophy observed in both species is considered to be a nonadverse adaptive response to high concentrations of drug in the tissue (Janas *et al.*, 2018), resulting from the frequent dosing and the high toxicological doses used in these studies. The same is valid for the observed increase in liver weight and pale appearance of the liver in the NHP. In the rat, the adaptive changes in the liver are associated with relatively small changes in ALT and ALP, but not AST, GGT, or bilirubin, mean that these findings have been classed as nonadverse.

One big contrasting finding between species was the overall distribution of SLN360 into the kidney and liver. In rats, a single dose of 10 mg/kg SLN360 resulted in approximately 2-fold greater levels in the kidney compared with the liver, a phenomenon that was compounded when multiple doses in a short timeframe were given at the higher doses (10 and 30 mg/kg). At the pharmacologically relevant 3 mg/kg dose, this distribution remained at a 2-fold difference, even after 5 weekly doses (kidney 144 µg/g tissue; liver 67 µg/g tissue). The ASGPR has a finite capacity for uptake and above certain doses, it is possible to saturate the receptor (Bon *et al.*, 2017; Debacker *et al.*, 2020). Therefore, one explanation for this discrepancy is that there is saturation of liver uptake mechanisms in the rat at higher doses, leading to a greater proportion of SLN360 entering the kidney over time. This is supported by the greater than dose-proportional increase in plasma SLN360 observed between the 3 and 10 mg/kg dose. This contrasted with the findings in the NHP that were dosed at higher levels, where liver levels were 2-fold that in the kidney even at the highest dose on day 30, with nearly full elimination of SLN360 observed in the kidney during the recovery period compared with the liver. It is of note that the toxicological studies with SLN360 were conducted using an aggressive weekly dosing regimen whereas clinical repeat dosing is projected at much longer dose intervals and at lower dose levels. Nevertheless, the toxicological target organs identified in the nonclinical safety evaluations of SLN360, ie, liver and kidneys, are amenable to routine monitoring in clinical studies and therefore the associated risk at pharmacological dose levels of SLN360 is considered low.

Additionally, differences in SLN360 plasma levels of up to 20% were observed between males and females in the rat. One potential explanation is that this difference is due to faster uptake into the kidney and/or liver in female versus male rats rather than a “true” accumulation as a consequence of dosing. This would require further investigation to confirm if this was the cause for this observation.

The cynomolgus monkey was confirmed to be a relevant PD species showing significant knockdown with an almost complete absence of *LPA* mRNA with a concomitant absence of serum Lp(a) even after 8 weeks recovery in NHP, with no adverse sequelae. Additionally, the absence of Lp(a) did not result in any changes to other blood chemistry parameters. These observations are similar to findings in humans where, based on published literature, pharmacologically induced reduction of Lp(a) to low levels, or even complete reduction to undetectable levels, is likely to have no detrimental effects in humans. It has been shown that low levels of Lp(a) are not detrimental to human health but in fact associate strongly with lower risk of cardiovascular diseases (Emdin *et al.*, 2016). Notably, the complete absence of circulating Lp(a) appears to have no effect on increasing morbidity or mortality (Emdin *et al.*, 2016; Lim *et al.*, 2014). Additionally, direct pharmacological reduction of Lp(a) through gene silencing has been evaluated clinically and does not appear to be associated with any safety concerns due to Lp(a) reduction (Koren *et al.*, 2022; Tsimikas *et al.*, 2015, 2020; Viney *et al.*, 2016).

When using traditional allometric scaling, the NOAEL of 200 mg/kg derived from the cynomolgus monkey results in a human equivalent dose of 64.5 mg/kg. When applying a 10-fold safety factor the maximum recommended safe starting dose would be 6.45 mg/kg. The flat starting dose of 30 mg in the first in human clinical study (Nissen *et al.*, 2022) is >10-fold below the maximum recommended safe starting dose and, therefore, considered to be suitable. Using a similar approach with the rat NOAEL at 30 mg/kg, the proposed 30 mg dose in a 60 kg subject (0.5 mg/kg) is the maximum recommended safe starting dose of 0.5 mg/kg based on the rat data.

The nonclinical profiles of GalNAc-siRNA molecules have been described extensively in the literature (Berman *et al.*, 2014, 2016; Henry *et al.*, 2016; Janas *et al.*, 2016, 2018). Data up to now suggest that there are specific effects that can be attributed to the platform and the toxicological effects are largely predictable. A body of evidence is accumulating that demonstrates the lack of genotoxicity of these molecules, while early immune activation issues are largely overcome with reduced PS content and the use of modified bases. Aside from any potential exaggerated pharmacology, histopathological findings are largely restricted to the liver, kidney and reticuloendothelial system (Janas *et al.*, 2018). The nonclinical safety evaluation of SLN360 has not revealed any unexpected findings when considering the data from other, structurally highly related emerging siRNA nucleic acid therapeutics that have already successfully completed phase 3 trials and been approved for clinical use, such as givosiran, inclisiran, and lumasiran (Balwani *et al.*, 2020; Garrelfs *et al.*, 2021; Ray *et al.*, 2020). Overall, the nonclinical safety profile of SLN360 supports the clinical evaluation of SLN360.

## SUPPLEMENTARY DATA

Supplementary data are available at *Toxicological Sciences* online.

## Supplementary Material

kfac067_Supplementary_Data
