# Supplementary material for: Preclinical Toxicological Assessment of A Novel siRNA, SLN360, Targeting Elevated Lipoprotein (a) in Cardiovascular Disease
Source: Toxicol Sci. 2022 Jun 23;189(2):237–49. doi: 10.1093/toxsci/kfac067 (PMC9516055; doi:10.1093/toxsci/kfac067)
Supplement: kfac067_Supplementary_Data [file kfac067_supplementary_data.pdf]

Pre-clinical Toxicological Assessment of A Novel siRNA, SLN360, Targeting Elevated Lipoprotein (a) in Cardiovascular Disease

David Rider, Simon Chivers, Julia Aretz, Mona Eisermann, Kathrin Löffler, Judith Hauptmann, Eliot Morrison, Giles Campion

Supplementary Material

Contents:

Supplemental Methods

Supplementary Table 1

Supplementary Table 2

Supplementary Table 3

Supplementary Table 4

Supplementary Table 5

Supplementary Figure 1

Supplementary Figure 2

Whole blood in vitro cytokine production

Peripheral blood from cynomolgus macaque and human donors (n=4 from each species) was collected by venipuncture and transferred into sterile plastic tubes containing anti-coagulant. Blood was used on the same day as it was collected. Blood (180µL) was transferred into 96-well U-bottom plates and 20µL of vehicle (0.9% sodium chloride), SLN360 (final concentration 10µM) or lipopolysaccharides from *Escherichia coli* serotype 0127:B8 (LPS, final concentration 50ng/mL; Sigma Aldrich Cat. No. L4516). Assay plates were incubated for 0, 2, 6 and 24h in an incubator at 37°C, 5.0% CO_2. At the end of incubation, assay plates were centrifuged at 400 g for 5 minutes at ambient temperature. Plasma was then collected into fresh V bottom plates and centrifuged at 400 g for 5 minutes at ambient temperature. Supernatants were collected into clean labelled tubes and stored at -80°C until cytokine analysis. Cytokines were analysed on a Bio-Plex 200 luminex system using the following kits: Human Magnetic Bead Custom Multiplex Kit ThermoFisher (PPX-09-MXFVMCV) and Non-Human Primate Magnetic Bead Custom Multiplex Kit ThermoFisher (PPX-09-MXGZFWT).

Zimmerman rabbit immunization and bleeding

SLN360 was conjugated to LPH (hemocyanin from *Limulus polyphemus* hemolymph, Sigma-Aldrich, Cat. No. H1757) via a maleimide coupling method for immunization purposes. For ELISA testing, a second conjugate was prepared (SLN360-BSA) using BSA (Bovine Serum Albumin, Sigma-Aldrich, Cat. No. A3059) as a carrier protein to avoid a false titer signal.

LPH-conjugated SLN360 was prepared in adjuvant (lipo-polysaccharides of the blue-green algae *Phormidium spec*, mineral oil, Tween 80 and Span 80) as a 3:1 mix adjuvant:SLN360-LPH and mixed until a stable emulsion was formed. Three female rabbits (Zimmerman breed) were immunized intramuscularly (i.m.) with the antigen conjugate St12-L4-LPH on day 1 (1.0mg), 8, 15, 57, 71 (0.5mg) and 99 (0.25mg). At designated timepoints (days 29, 78, 113) blood was collected by puncture of the *A. auriculares* in 50 mL centrifuge tubes (Falcon, TPP). The blood was coagulated at room temperature for four hours and then stored overnight at 2-8° C. Antiserum was extracted by centrifugation (20 min at 4000xg, twice). A preservative (0.1% ProClin 300) was added, and the antiserum was stored at 2-8° C for possible purification purposes.

Anti-drug antibody Titer determination

Non-competitive ELISA

ELISA microtiter plates (96 well, 1x8 F-strips, high binding capacity, Greiner Bio-One, Cat. No. 762071) were coated with 100 µL/well of antigen solution (10 µg/mL) in coating buffer (50 mM $NaHCO_3$, pH 9.5) overnight. The plates were emptied and blocked with 200 µL/well TBS-TW (Blue Cap Solution 10x Wash Buffer, Art-No: S210.500) + 1 % fetal calf serum (FCS) for thirty minutes under continuous shaking and washed thrice with 250 µL/well TBS-TR. All sera were diluted in TBS-TW + 1 % FCS in a threefold dilution series from 1:100 up to 1:218,700. One hundred µL of the dilutions were added to the wells of the plates, each in duplicate. TBS-TW + 1 % FCS were used as assay blank. The plates were incubated for 1 h under continuous shaking

and washed four times with 250 µL/well of TBS-TW. One hundred µL of the specific anti-rabbit-IgG-HRP conjugate (purified polyclonal rabbit antibody, Sigma®, Cat. No. A4914), diluted 1:30,000 in TBS-TW, were added to the wells of the plates. The plates were incubated for 1 h under continuous shaking and washed four times with 250 µL/well TBS-TW. The colorimetric enzyme reaction for end point titration was carried out by adding 100 µL of TMB-ONE (Ready to use) to the wells of the plates. The enzyme reaction was stopped after 15 min by adding directly 100 µL/well of 0.5 M sulfuric acid. The optical density at 450 nm (OD450) was measured with a multichannel microplate reader ELx808 by Bio-Rad and the raw data were obtained through the corresponding software Gen5™.

Competitive ELISA

To avoid false positive titer signals against the SLN360-BSA-conjugate, the obtained sera of bleedings from days 29 and 78 was preincubated with 100 µg/mL unconjugated antigen for 15 minutes at room temperature. The non-competitive ELISA protocol was then followed as described above.

Human ether-a-go-go related gene (hERG) tail current measurements

Interaction of SLN360 with the hERG channel was examined in HEK293 cells stably expressing this potassium channel. Cells were seeded onto 35 mm sterile culture dishes containing 2 mL culture medium (1:1 mixture of Dulbecco's modified eagle medium and nutrient mixture F-12 (D-MEM/F-12 1x, liquid, with L-Glutamine) supplemented with 10% fetal bovine serum). SLN360 was dissolved directly in bath solution (Sodium Chloride 137 mM, Potassium Chloride 4 mM, Calcium Chloride 1.8 mM, Magnesium Chloride 1 mM HEPES 10 mM, D-Glucose 10 mM pH (NaOH) 7.4) to prepare a stock solution of 20mg/mL with a final in assay concentration of 20µg/mL.

Cells were seeded at a density allowing single cells to be recorded in 35mm culture dishes and were placed on the dish holder of the microscope and continuously perfused (at approximately 1 mL/min) with the bath solution. All solutions applied to cells including the pipette solution (Potassium Chloride 130 mM, Magnesium Chloride 1 mM, Mg-ATP 5 mM, HEPES 10 mM, EGTA 5 mM, pH (KOH) 7.2) were maintained at room temperature. Shortly before entering the recording chamber, the test solutions were heated to 36 ± 1°C. After formation of a Gigaohm seal between the patch electrodes and individual hERG stably transfected HEK293 cells the cell membrane across the pipette tip was ruptured to assure electrical access to the cell interior (whole-cell patch configuration). As soon as a stable seal was established, hERG outward tail currents were measured upon depolarization of the cell membrane to +20 mV for 2 s (activation of channels) from a holding potential of -80 mV and upon subsequent repolarization to -40 mV for 3 s. This voltage protocol (as shown below) was run at least 10 times at intervals of 10 s. If current density was judged to be too low (< 500 pA) for measurement, another cell was recorded. Once control recordings were accomplished (less than 5% change of current amplitude within 100 s), cells were continuously perfused with the test solutions as detailed in section 3.7, bath solution (> 255 s perfused) or 100 nM E-4031. During wash-in of the test item, the voltage protocol indicated above ran continuously again at 10 s intervals until the steady-state level of current block was reached.

Using SigmaPlot 11.0, the recorded current amplitudes at the steady state level of current inhibition were compared to those from control conditions measured in the pre-treatment phase of the same cell. Data from three individual experiments were collected and the corresponding mean values and standard errors calculated.

To determine whether the observed current inhibition was due to a test item interaction with the hERG channel or due to current rundown, these residual currents were compared to those measured in bath solution only. As only one concentration was tested, the statistical significance was tested using Student's T-Test (GraphPad Prism 5).

Genotoxicity Assessment

A standard battery of recommended test was performed and analyzed according to ICH guideline S2 (R1) on genotoxicity testing and data interpretation for pharmaceuticals intended for human use.

AMES test

SLN360 was assayed for mutation in five histidine-requiring strains (TA98, TA100, TA1535, TA1537 and TA102) of Salmonella typhimurium, both in the absence and in the presence of metabolic activation by an Aroclor 1254-induced rat liver post-mitochondrial fraction (S-9). SLN360 was prepared in 0.9% saline with final in-assay concentrations ranging from 5-5000µg/plate. Bacterial strains were plated in triplicate in 2mL molten top agar, 0.1mL bacterial culture, 0.1mL SLN360 or 0.05mL positive control compound, 0.5mL 10% S-9 mix (Sodium phosphate buffer pH 7.4 100µmoles, Glucose-6-phosphate (disodium) 5 µmoles, β-Nicotinamide adenine dinucleotide phosphate (disodium) 4 µmoles, $MgCl_2$ 8 µmoles, KCl 33 µmoles, d.H_2O to volume, S-9 100 µL) or buffer solution (100µmoles Sodium phosphate buffer pH 7.4) followed by rapid mixing and pouring on to Vogel-Bonner E agar plates. When set, the plates were inverted and incubated protected from light for 3 days in an incubator set to 37°C. Following incubation, these plates were examined for evidence of toxicity to the background lawn, and where possible revertant colonies were counted. Counting was performed electronically using a Sorcerer Colony Counter (Perceptive Instruments). Individual plate counts were recorded separately, and the mean and standard deviation of the plate counts for each treatment were determined.

In Vitro L5178Y Gene Mutation Assay at the *tk* locus

SLN360 was assayed for the ability to induce mutation at the *tk* locus (5-trifluorothymidine [TFT] resistance) in mouse lymphoma cells using a fluctuation protocol in the absence and presence of metabolic activation by an Aroclor 1254-induced rat liver post-mitochondrial fraction (S-9). SLN360 was formulated in 0.9% saline with final in-assay concentrations ranging from 1.0-500µg/mL. Control compounds (methyl methane sulphonate [MMS] and benzo[a]pyrene {B[a]P}) were prepared in dimethyl sulphoxide with final in assay concentrations of 5.0 and 7.5µg/mL (24h) or 7.5 and 15.0µg/mL (3h) for MMS, or 2.0 and 3.0µg/mL (3h) for B[a]P. For all treatments 2 mL vehicle, test article, culture medium for the untreated control or 0.2 mL positive control solution (plus 1.8 mL of 0.9% saline) was added. Treatments were carried out in duplicate both in the absence and presence of S-9 by addition of either

150 mM KCl or 10% S-9 mix respectively. The final S-9 volume in the test system was 1% (v/v).

For 3-hour treatments in the absence and presence of S-9, at least 10^7 cells in 18 mL tissue culture medium (cells in RPMI 10 diluted with RPMI A to give a final concentration of 5% serum) were placed in an incubator set to 37°C with gentle agitation for 3 hours. For 24-hour treatment in the absence of S-9 at least 4 x 10^6 cells in 18 mL RPMI 10 were cultured in tissue culture flasks with 5% v/v CO_2 in air at 37°C with no agitation (static incubation) for 24 hours. Cultures were centrifuged (200 g) for 5 minutes, washed with the appropriate tissue culture medium, centrifuged again (200 g) for 5 minutes, and then resuspended in RPMI 10 medium at a density of 2 x 10^5 cells/mL.

Cells were transferred to tissue culture flasks for growth throughout the expression period (48h). At the end of the expression period, cell concentrations in the selected cultures were adjusted to give 1 x 10^4 cells/mL for plating for TFT resistance. TFT (300 µg/mL) was diluted approximately 100-fold into these suspensions to give a final concentration of 3 µg/mL. Cell suspension (0.2 mL) was placed into each well of four 96-well microtiter plates (2 x 10^3 cells/well). Plates were placed in a humidified incubator at 37°C with 5% v/v CO_2 in air until scoreable (12 to 13 days) and wells containing clones were identified and counted. In addition, the number of wells containing large colonies and the number containing small colonies were scored for the negative and positive controls.

In Vitro Human Lymphocyte Micronucleus Assay

SLN360 was tested in an in vitro micronucleus assay using duplicate human lymphocyte cultures prepared from the pooled blood of two male donors in a single experiment. Treatments covering a broad range of concentrations, separated by narrow intervals, were performed both in the absence and presence of metabolic activation (S-9) from Aroclor 1254-induced rats. The test article was formulated in 0.9% saline and the highest concentration tested in the Micronucleus Experiment was 500 µg/mL. Positive control compounds had final in assay concentrations of 0.3µg/mL (mitomycin c 3h, no S-9), 3.0-7.0µg/mL (cyclophosphamide, 3h, with S-9) and 0.04µg/mL (vinblastine, 24h, no S-9).

For each experiment, an appropriate volume of whole blood was drawn from the peripheral circulation into heparinized tubes on the same day as culture initiation. Blood was used immediately and pooled using equal volumes from each donor prior to use. Whole blood cultures were established in sterile disposable centrifuge tubes by placing 0.4 mL of pooled heparinized blood into 7.6 mL pre-warmed (in an incubator set to 37°C) HEPES-buffered RPMI medium containing 10% (v/v) heat inactivated FCS and 0.52% penicillin / streptomycin. Blood cultures were placed in an incubator set to 37°C for approximately 48 hours and rocked continuously. Then 1mL of S-9 mix or KCl and 1mL of SLN360 or vehicle were added for a final volume of 10mL. For positive control cultures, 0.9mL culture medium and 0.1mL positive control were added. The mitogen phytohemagglutinin (PHA, reagent grade) was included in the culture medium at a concentration of approximately 2% of culture to stimulate the lymphocytes to divide.

In vivo rat bone marrow micronucleus test

All positive control animals (20mg/kg cyclophosphamide in 0.9% saline) and five toxicity animals/sex/group from the 29 day repeat dose toxicity study in the rat were subjected to micronucleus assessment. All animals were sampled approximately 24 hours after the final dosing for bone marrow. One femur from each animal was processed and analyzed.

Bone marrow was flushed from the femur of each animal with fetal bovine serum. Additional fetal bovine serum was added to each sample prior to filtration through cellulose columns. Cells were pelleted by centrifugation, and the supernatant decanted and pellet re-suspended in serum. Centrifugation was then repeated, and the majority of supernatant decanted. The pellet was gently re-suspended in the remaining serum and at least three slides were prepared, air dried, and fixed in absolute methanol. At least one slide for each animal was rinsed in distilled water, then stained in 12.5 µg/mL acridine orange. Stained slides were rinsed in phosphate buffer, air dried, and stored in the dark at room temperature prior to analysis.

Scoring was carried out using fluorescence microscopy at an appropriate magnification. All slides were allocated a random code and analyzed, under blinded conditions, by an individual not connected with the dosing phase of the study.

Initially the relative proportions of polychromatic erythrocytes (PCE), seen as bright orange enucleate cells, and normochromatic erythrocytes (NCE), seen as smaller dark green enucleate cells, were determined until a total of at least 500 cells (PCE plus NCE) had been analyzed. Then at least 4000 PCE/animal were examined for the presence of MN. The following were then calculated: %PCE for each animal and the mean for each group, frequency of MN PCE (i.e., MN per number of PCE scored) and %MN PCE for each animal and the group mean %MN PCE (±standard deviation).

The percentage of MN PCE in each treated group were compared with the vehicle control using a Wilcoxon Rank Sum test. The tests were interpreted with one-sided risk for increased frequency with increasing dose for percentage of MN PCE. The Terpstra Jonckheere test for dose response was also performed. Probability values of $p \leq 0.05$ were accepted as significant.

The induction of clastogenic / aneugenic damage was considered to have occurred if: 1) A statistically significant increase in the frequency of MN PCE occurred at one or more dose levels; 2) The incidence and distribution of MN PCE exceeded the laboratory's historical vehicle control data; 3) A dose-response trend in the proportion of MN PCE (where more than two dose levels were analyzed) was observed. Results were considered positive in this assay if all of the above criteria were met.

Supplementary table 1 – Primer sequences

Name	Primer/ Probe	Sequence 5' to 3'
hACTB	UPR	GCATGGGTCAGAAGGATTCCTAT
	PRB	YY-TCGAGCACGGCATCGTCACCAA-BHQ1
	LWR	TGTAGAAGGTGTGGTGCCAGATT
cACTB	UPR	AAGGCCAACCGCGAGAAG
	PRB	YY-TGAGACCTTCAACACCCCAGCCATGTAC-BHQ1
	LWR	AGAGGCGTACAGGGACAGCA
hcPPIB	UPR	AGATGTAGGCCGGGTGATCTTT
	PRB	YY-TGTTCCAAAAACAGTGGATAATTTTGTGGCC-BHQ1
	LWR	GTAGCCAAATCCTTTCTCTCCTGT
APOB	UPR	TCATTCCTTCCCCAAAGAGACC
	PRB	FAM-CAAGCTGCTCAGTGGAGGCAACACATTA-BHQ1
	LWR	CACCTCCGTTTTGGTGGTAGAG
PLG	UPR	TTCACCACCGACCCCAAC
	PRB	FAM-TGCACAACACCTCCACCATCTTCTGG-BHQ1
	LWR	TTCACCTGTTCCCTTCAGACAC
hLPA	UPR	TGGCAGCCCCTTGGTG
	PRB	FAM-ACGATGCTCAGATGCAGAATGGACTGC-BHQ1
	LWR	AGAATAACATTCGGAGGGACGA
cLPA	UPR	GTGTCCTCGCAACGTCCA
	PRB	FAM-TGGCTGTTTCTGAACAAGCACCAATGG-BHQ1
LPAL2	UPR	CGCCACATCAGCACATGG
	PRB	FAM-CCTGACCAGGAACTACTGCAGGAATCCA-BHQ1
	LWR	ACACCAGGGTTGTTTCCCAGA
ADAM10	UPR	TCCAAAGTTGCCTCCTCCTAAA
	PRB	FAM-AAGAGGAGGAGACCTCCACAGCCCATT-BHQ1
	LWR	TGACGCTGGGGTTGCTG
AGAP1	UPR	GGCTGAATGTGGAGAGGGTCTT
	PRB	FAM-CCAGGACGTTGCCCAGAAGATTGTTG-BHQ1
	LWR	GGAATGGCTGGGAGAATTAGGT
SFT2DT	UPR	GGACTTGCACTTATCTTCTGCATTT
	PRB	FAM-TGGCATTGACGTGGTACAGCCTTTCC–BHQ1
	LWR	CACGGCAAAACACTTCTTCACA
TAF15	UPR	GGACAAAGTTATTCACAGTCCTATGGT
	PRB	FAM-ACCAGGGACAGCAGCAAAACATGGAAT-BHQ1
	LWR	TCTTCCACCTTGGCTTCCTG
VCAN#1	UPR	AGGCACGGCAATCTATTTACCA
	PRB	FAM-AATGAACCCGTGCCTTAACGGAGGC-BHQ1
	LWR	CAGGTGCATACGTAGGAAGTTTCA
VCAN#2	UPR	TCCCTGCAATTACCATCTCACC
	PRB	FAM-CAGCCCCCTGTTGTAGAAAATGCCAAG-BHQ1

A = Adenine; *ACTB* = Beta actin; *ADAM10* = Disintegrin and metalloproteinase domain-containing protein 10; *AGAP1* = Arf-GAP with GTPase, ANK repeat and PH domain-containing protein 1; BHQ1 = BlackHoleTM Dark Quencher; C = Cytosine; c = specific for cynomolgus; FAM = fluorescein derivative; G = Guanine; h = specific for human; *LPA* = apolipoprotein (a); *LPAL2* = Lipoprotein(a) like 2, pseudogene; LWR = lower primer; *PLG* = plasminogen; *PPIB* = Peptidylprolyl Isomerase B; PRB = probe; *SFT2DT* = Vesicle transport protein SFT2B; T = Thymine; *TAF15* = TATA-box binding protein associated factor 15; UPR = upper primer; *VCAN* = Versican core protein; YY = Yakima Yellow.

Supplementary Table 2 – organs assessed in 29-day toxicology studies

Tissue / organ	NHP	Rat	Tissue / organ	NHP	Rat
Adrenal	+	+	Mandibular Salivary Gland	+	+
Aorta	+	+	Muscle, Biceps Femoris	+	+
Brain	+	+	Nerve, Optic[a]	+	+
Caecum	+	+	Nerve, Sciatic	+	+
Colon	+	+	Ovary	+	+
Dosing sites	+	+	Oviduct	+	
Duodenum	+	+	Pancreas	+	+
Epididymis	+	+	Pituitary	+	+
Esophagus	+	+	Prostate	+	+
Eye[a]	+	+	Rectum	+	+
Femur + Marrow	+	+	Seminal Vesicle	+	+
Gall Bladder	+		Skin/Subcutis	+	+
GALT/Peyers Patch	+	+	Spinal Cord, Cervical	+	+
Gross lesions (variable)	+	+	Spinal Cord, Lumbar	+	+
Heart	+	+	Spinal Cord, Thoracic	+	+
Ileum	+	+	Spleen	+	+
Jejunum	+	+	Sternum + Marrow	+	+
Kidney	+	+	Stomach	+	+
Liver	+	+	Testis[a]	+	+
Lung	+	+	Thymus	+	+
Lymph Node, Axillary	+	+	Thyroid and Parathyroid	+	+
Lymph Node, Inguinal		+	Tongue	+	+
Lymph Node, Mandibular	+	+	Trachea	+	+
Lymph Node, Mesenteric	+	+	Urinary Bladder	+	+
Mammary Gland	+	+	Uterus	+	+

All tissues were fixed in neutral buffered 10% formalin unless specified otherwise. [a]Tissue fixed with Davidson's fixative. + = examined

Supplementary Table 3 – off targets across species

Species	Predicted # of mismatches to SLN360 antisense	Predicted Off-target Gene	Liver expressed	Transcript(s)
**Human**	19	***LPAL2***	yes	pseudogene: NR_028092.1, NR_028093.1
	18	*LPAL2*	yes	NR_028092.1, NR_028093.1
	17	*LPAL2*	yes	NR_028092.1, NR_028093.1
	16	***VCAN***	low	ENST00000265077.8
	16	*ADD2*	no	*not analysed*
	16	***ADAM10***	yes	ENST00000260408.8
	16	***AGAP1***	yes	ENST00000304032.13
	16	***SFT2D2***	yes	ENST00000271375.7
	16	***TAF15***	yes	not in reference gene (29 transcript variants)
**Cynomolgus**	17	*LOC102146523*	n/a	predicted gene: XR_001489902.1
	16	*PCNX1*	yes	ENSMFAT00000035088.1
	16	*ADD2*	no	*not analysed*
	16	*LOC102129109*	n/a	XR_001490826.1
	16	*ULK4*	yes	XM_005546755.2 (1/10 transcripts)
	16	*TAF15*	yes	XM_005583483.2
	16	*LOC102146523*	n/a	XR_001489902.1, XR_001489901.1
**Mouse**	16	*Agap1*	yes	*not analysed*
	16	*Cyp2j5*	n/a	*not analysed*
	16	*Pex6*	yes	*not analysed*
	16	*Mme*	yes	*not analysed*
	16	*Mki67*	low	*not analysed*
**Rat**	16	*Agap1*	yes	ENSRNOT00000065720.1
	16	*Cxcl16*	yes	ENSRNOT00000032926.4
	16	*Slfn5*	yes	ENSRNOT00000076133.1
	16	*Mki67*	low	ENSRNOT00000038176.5

ADAM10 = Disintegrin and metalloproteinase domain-containing protein 10; *ADD2* = Adducin 2; *AGAP1* = Arf-GAP with GTPase, ANK repeat and PH domain-containing protein 1; *Cxcl16* = C-X-C Motif Chemokine Ligand 16; *Cyp2j5* = Cytochrome P450 2J5; *LPAL2* = Lipoprotein(a) like 2, pseudogene; *Mki67* = Marker of proliferation Ki-67; *Mme* = Membrane metalloendopeptidase; *PCNX1* = Pecanex 1;*SFT2DT* = Vesicle transport protein SFT2B; *Slfn5* = Schlafen family member 5;*TAF15* = TATA-box binding protein associated factor 15; *ULK4* = Unc-51 like kinase 4; *VCAN* = Versican core protein;

Supplementary Table 4 – Total bilirubin and gamma-glutamyl transferase levels in the rat 29-day toxicology study

		T.BI (µmol/L)			GGT (IU/L)		
Group	Sex	Mean	SD	n	Mean	SD	n
1[a]	M	<1.7	0	10	<3	0	10
	F	<1.7	0	10	<3	0	10
2[a]	M	<1.7	0	10	<3	0	10
	F	<1.8	0.18	10	<3	0	10
3[a]	M	<1.7	0.04	10	<3	0	10
	F	<1.8	0.19	10	<3	0	10
4[a]	M	<2.0	0.46	10	<3	0	10
	F	<1.8	0.18	10	<3	0	10
1 (Rec)	M	<1.7	0.09	5	<3	0	5
	F	<1.7	0	5	<3	0	5
4 (Rec)	M	<1.7	0	4	<3	0	4
	F	<2.0	0.39	5	<3	0	5

[a]Samples were taken on day 30, 24 h post-final dose. F – Female; GGT – Gamma-Glutamyl Transferase; M – Male; Rec – Recovery animals; SD – Standard Deviation; T.BI – Total Bilirubin

Supplementary Table 5 – Total bilirubin levels in the NHP 29-day toxicology study

			T.BI (μmol/L)		
Group	Sex	Timepoint	Mean	SD	n
1	M	Pre-dose	<1.8	0.22	5
		Day 25[a]	<3.2	2.55	5
		Day 46	3.4	2.26	2
	F	Pre-dose	2.3	0.41	5
		Day 25	3.1	1.43	5
		Day 46	3.0	1.77	2
2	M	Pre-dose	<2.0	0.26	3
		Day 25	2.5	0.15	3
	F	Pre-dose	<1.7	0.00	3
		Day 25	2.2	0.3	3
3	M	Pre-dose	<3.0	1.89	3
		Day 25	<2.1	0.75	3
	F	Pre-dose	<1.9	0.35	3
		Day 25	<1.9	0.4	3
4	M	Pre-dose	<2.1	0.62	5
		Day 25	<2.1	0.63	5
		Day 46	2.0	0.28	2
	F	Pre-dose	<2.5	1.53	5
		Day 25	<1.8	0.13	5
		Day 46	<1.7	0.00	2

F – Female; M – Male; NHP – Non-Human Primate; SD – Standard Deviation; T.BI – Total Bilirubin

Supplementary Figure 1

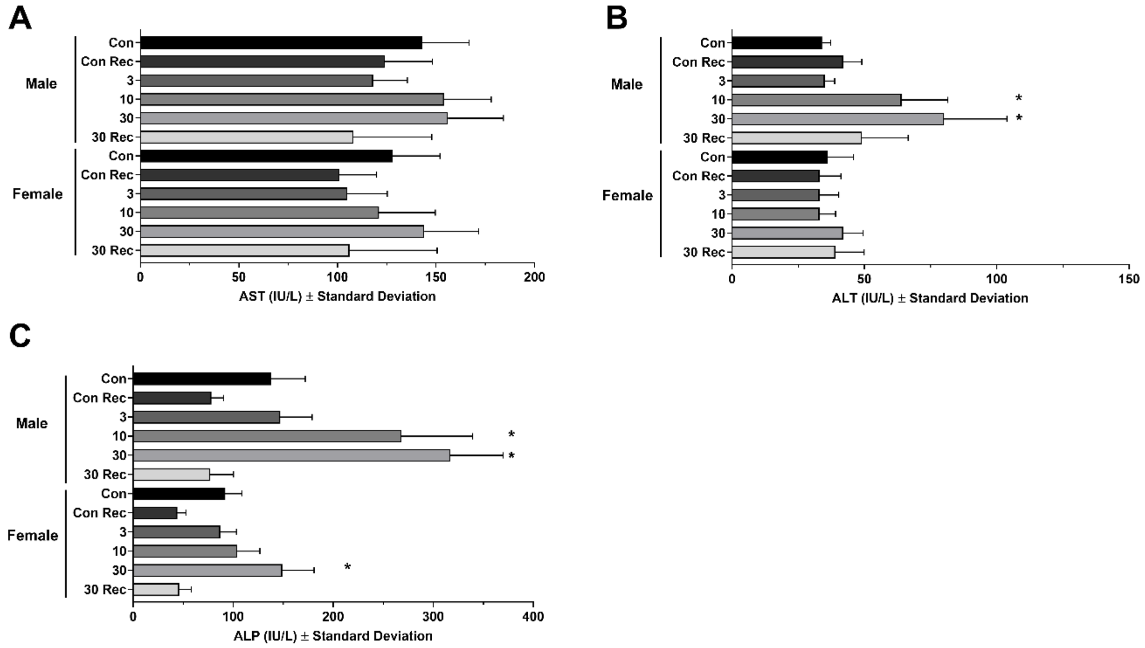

Effect of SLN360 on liver enzyme activity in rats from the 29-day GLP toxicology study

Blood samples were taken to measure liver enzyme activity (A) AST, (B) ALT and (C) ALP on day 30 (24h post-final dose) and post-recovery. *p<0.05 as determined using a one-way ANOVA with Dunnett's post-hoc analysis. Significant increase in ALT and ALP activity were observed at 10 and 30 mg/kg in males and for ALP at 30mg/kg in females at day 30. These changes were no longer evident following the recovery period. ALP – Alkaline Phosphatase; ALT – Alanine Aminotransferase ; AST – Aspartate Aminotransferase; Con – Control animals; Rec – Recovery Animals.

Supplementary Figure 2

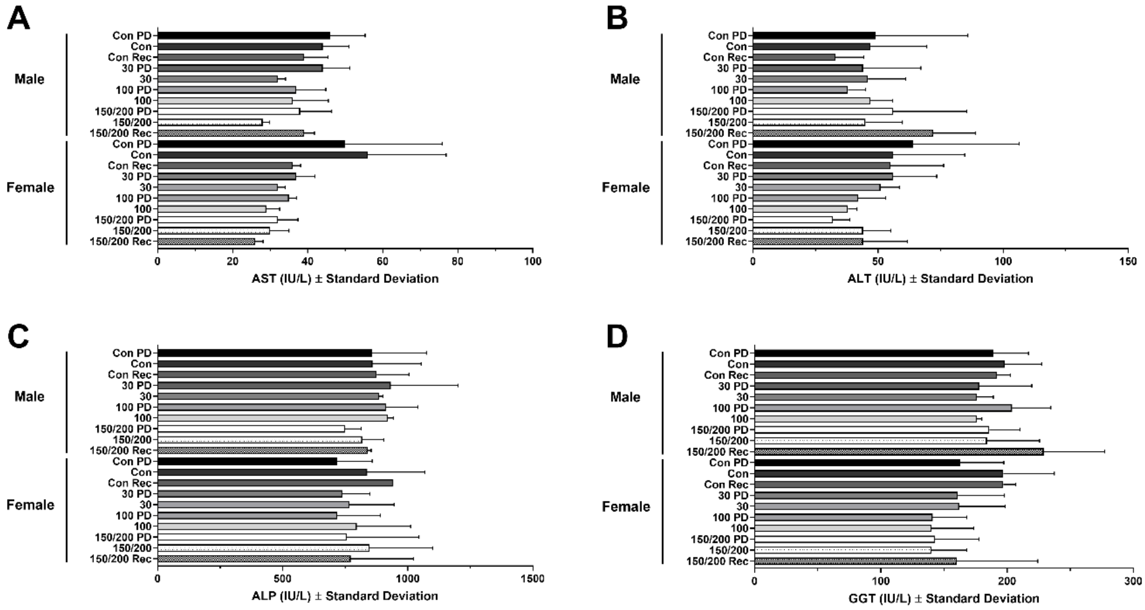

Effect of SLN360 on liver enzyme activity in NHP from the 29-day GLP toxicology study

Blood samples were taken to measure liver enzyme activity (A) AST, (B) ALT, (C) ALP and (D) GGT on day 25 and during recovery (day 46). Data was analyzed using a one-way ANOVA with Dunnett's post-hoc analysis. No significant increases in enzyme activity were observed in SLN360 -treated animals at day 25. No analysis was made on recovery animals due to limited animal number. ALP – Alkaline Phosphatase; ALT – Alanine Aminotransferase ; AST – Aspartate Aminotransferase; Con – Control animals; GGT – Gamma-Glutamyl Transferase; PD – Pre-Dose; Rec – Recovery Animals.
